# B Cell Activating Factor, Renal Allograft Antibody-Mediated Rejection, and Long-Term Outcome

**DOI:** 10.1155/2018/5251801

**Published:** 2018-06-06

**Authors:** Haiyan Xu, Xiaozhou He, Renfang Xu

**Affiliations:** Urology Department, Third Affiliated Hospital of Soochow University, Changzhou 213003, China

## Abstract

Antibody-mediated rejection (ABMR) of renal allograft lacks typical phenotypes and clinical manifestations, always resulting in delayed diagnosis and treatment. It has been considered to be an elemental factor influencing the improvement of the long-term outcome of renal allograft. The B cell activating factor (BAFF) signal plays a fundamental function in the process of antibody-mediated immune response. Data from recipients and the nonhuman primate ABMR model suggest that the BAFF signal participates in the ABMR of renal allograft, while there are objections. The challenges in the diagnosis of ABMR, different study population, and details of research may explain the discrepancy. Large quantities of dynamic, credible data of BAFF ligands and their association with renal allograft pathological characteristics would constitute a direct proof of the role of BAFF in the progression of renal allograft ABMR.

## 1. Introduction

New immunosuppressive reagents and gradual improvements in posttransplantation management have fostered great improvements in the short-term outcomes of renal transplantation. However, long-term outcomes have not seen similar improvements for several reasons, of which antibody-mediated rejection (ABMR), most notably chronic active ABMR, should be the elemental one [1, 2].

It is now recognized that there are two types of renal allograft ABMR [3, 4]. Type 1 ABMR results from persistence, a resurgence of preexisting donor-specific antibodies (DSA), or both in sensitized patients and usually occurs early posttransplantation. Type 2 ABMR is associated with de novo DSA and usually occurs more than one year posttransplantation. Some acknowledged risk factors may predict the risk of onset of type 1 ABMR, which facilitates timely and effective treatment. There is an absence of characteristic markers and clinical manifestations of type 2 ABMR [5], which is a significant contributor to late renal allograft loss.

## 2. Open Questions about Optimal Humoral Indicators

C4d was found to be a potential marker for ABMR in kidney allografts. Standardized scoring of C4d (the complement split product) staining in renal graft biopsies was set at the Banff meeting of 2003 [6]. However, multiple studies that followed strongly supported the existence of ABMR with negatively or minimal/equivocal C4d deposition within peritubular capillaries [7–9].

C4d is not an ideal humoral indicator associated with adverse kidney transplant outcomes; neither are DSA, IgG subclasses, and C1q-binding DSA [10–14]. It is important to find optimal markers or indicators of ABMR in renal allografts for improvements of the long-term outcomes of kidney transplantation.

## 3. B Cell Activating Factor (BAFF): An Optimal Marker for ABMR?

**Figure 1:**
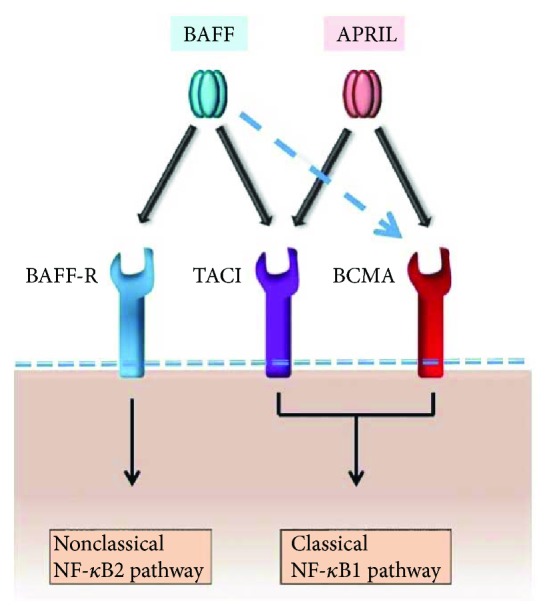
BAFF, APRIL, and their receptors; BAFF binds with high affinity to both BAFF-R and TACI but with weak affinity to BCMA; APRIL conversely binds TACI and BCMA but does not bind BAFF-R. BAFF-R ligation primarily results in the activation of the nonclassincal NF-*κ*B2 pathway, whereas TACI or BCMA ligation initiates the classical NF-*κ*B1 pathway. These downstream signaling cascades promote cell survival and growth.

**Figure 2:**
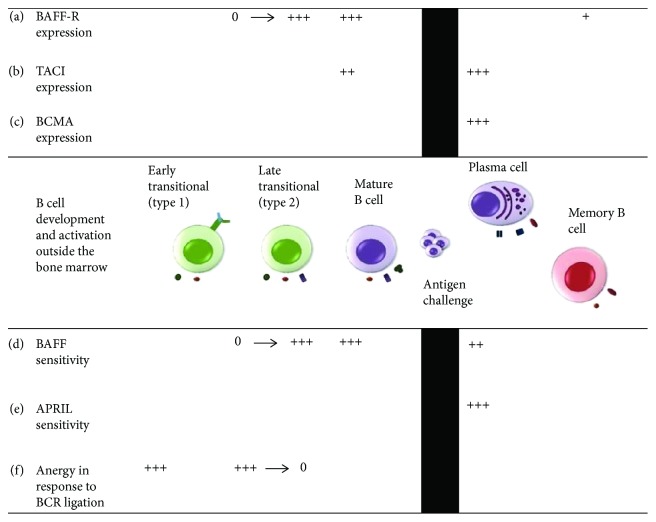
Expression of BAFF-R, BCMA, and TACI on B cells at different stages of development and activation. (a) BAFF-R begins to be expressed at the late transitional stage and is present on all mature B cells. Its expression is reduced on B cell entry into the GC reaction and is reexpressed on memory B cells but absent on plasmablasts and plasma cells. (b) TACI expression can be detected after the late transitional stage and also on plasmablasts and plasma cells. (c) BCMA expression is restricted to plasmablasts and plasma cells. (d and e) BAFF promotes the maturation of transitional B cells and the subsequent survival of mature B cells, whereas BAFF and APRIL can both promote plasma cell survival. Memory B cell survival and reactivation are independent of BAFF or APRIL signaling. (f) BAFF-R signals interplay with BCR signals in determining B cell maturation and survival. Under conditions of limiting BAFF availability, ligation of the BCR by antigen leads to anergy and reduced lifespan in immature transitional B cells.

B cell activating factor (BAFF, also known as BLys) shares high homology with the other ligand, APRIL. These two ligands bind to three receptors, BAFF-R, TACI, and BCMA. BAFF interacts with all three receptors, whereas APRIL can only interact with TACI or BCMA (Figure 1) [15]. Early work established that total systemic BAFF signals via BAFF-R are essential for the survival and selection of preimmune B cells (Figure 2) [15, 16]. Locally produced BAFF is involved in regulating aspects of humoral immune responses. The pattern of expression of the BAFF family receptors creates independent selective and homeostatic niches [17]. Abnormal BAFF signals have been confirmed in several autoimmune disorders, such as systemic lupus erythematosus, rheumatoid arthritis, and Sjogren's syndrome [18, 19].

Thaunat et al. first published findings about BAFF in renal transplantation. They found that BAFF provides survival signals to B cells and allows them to escape rituximab-induced apoptosis in tertiary lymphoid organs [20]. Our group also carried out some early related work. We found that cell-surface BAFF was significantly highly expressed on peripheral CD3^+^ T cells in recipients over the course of 5 years and in those with abnormal renal function and was significantly correlated with anti-HLA I and II antibodies [21, 22]. These results suggested that BAFF may be involved in the development of graft loss and influences the long-term outcome of kidney allografts. Further studies have shown BAFF to be highly expressed in acute ABMR tissues and chronic rejection tissues with high C4d deposition. There was a significant correlation between pathological traits and BAFF expression levels or C4d expression levels [23]. Thus, it was presumed that BAFF participates in humoral-mediated renal allograft rejection.

Our early conclusions were consistent with those of other research groups, who later published their observations about BAFF research in renal transplantation. Some of these studies were performed on adult recipients and some on pediatric patients [24–28]. Kwun et al. confirmed this from another perspective, and they observed that neutralizing BAFF/APRIL with TACI-Ig (atacicept) could prevent early DSA formation and ABMR development in a nonhuman primate ABMR model with T cell depletion [29]. These results provided convincing proof regarding the function of BAFF signals in the ABMR of renal allografts.

However, some research groups collected data that do not support this conclusion [30–33].

## 4. Sustained and In-Depth Research Be Encouraged

The challenges in diagnosis, grading, and staging of ABMR are complicated by the fact that morphological features are dependent on the time point in the course of the disease and that the dynamics of disease course show significant variability among individual patients. Different study populations were investigated, and different patients' inclusion criteria, sample sizes, and sampling times may explain the discrepancy. Large quantities of dynamic, credible data of BAFF ligands and their association with renal allograft pathological characteristics constitute direct proof of the role of BAFF in the progression of renal allograft ABMR. Multicenter combined studies should be encouraged. An optimal ABMR animal model of kidney transplantation would help resolve the difficulties in specimen acquisition. BAFF ligand detection combined with other indexes, for example, C4d, C1q-binding DSA, preexisting DSA, de novo DSA, and IgG subtypes, merits further exploration. BAFF could be a predictor of ABMR in kidney transplantation recipients, as predicted by Pongpirul et al. [34].

BAFF ligands are usually produced by myeloid cells, and the receptors are expressed by B lymphocytes. BAFF expression can increase in response to cytokine stimulation, and interferon-*γ*, interleukin-10, transforming growth factor-*β*, and granulocyte-clonal stimulator factor are all listed. Under some conditions, BAFF can also be found on some nonmyeloid cells, for example, epithelial cells, osteoclasts, and adipocytes [35–37]. However, little work has been devoted to the action of BAFF on nonmyeloid cells. In the context of kidney transplantation, an abnormally high expression of BAFF ligands and receptors has also been observed on tubular epithelial cells in renal allografts [38]. Whether they function in the biological activity of RTECs, what their mechanisms of action are, and what kind of role they play in AMBR remain unknown. Renal tubular epithelial cells are one kind of renal parenchymal cells, and they play an important role in the maintenance of renal allograft function. Resolving these issues would facilitate the mechanism underlying BAFF ligands and receptors in the progression of renal allograft ABMR.

## 5. Conclusion

The chronic humoral response is always insidious and sometimes mixed with T-cell-mediated rejection and nonspecific manifestations of chronic drug intoxication. Specific recipients can experience considerably different responses. However, there are always histologic, serologic, and molecular fingerprints indicating the occurrence and development of ABMR. Currently, the quality of detection reagents, cytokine techniques, cell subset analysis, and alloactivity detection are all very high. Advances in pertinent B cell research techniques may facilitate identification of markers for ABMR [39]. Other molecular detection and labelling techniques and established animal models would provide big convenience for further exploration of the BAFF ligands and receptors during the process of renal allograft ABMR. And sustained and effective investigation would finally improve the long-term outcome of renal transplantation.
